# Antiplatelet therapy for primary prevention of stroke in patients with carotid web: a prospective cohort study

**DOI:** 10.3389/fneur.2026.1955675

**Published:** 2026-10-07

**Authors:** Xiaomeng Wang, Pu Liu, Yi Hu, Yuzhu Zhang, Jingjiao Chen, Sijia Tian, Xiaosa Chi

**Affiliations:** 1 Department of Geriatric Medicine, The Affiliated Hospital of Qingdao University, Qingdao, China; 2 Department of Radiology, The Affiliated Hospital of Qingdao University, Qingdao, China; 3 Department of Neurology, Medical College of Georgia Augusta University, Augusta, GA, United States

**Keywords:** carotid web, cox regression with firth’s penalized maximum likelihood method, primary stroke prevention, prospective cohort study, single antiplatelet therapy

## Abstract

**Background:**

Carotid webs have emerged as an etiology for ischemic stroke in younger patients, with a high recurrence rate. However, the optimal prevention strategy for asymptomatic carotid webs remains uncertain.

**Methods:**

This prospective cohort study was conducted at the Affiliated Hospital of Qingdao University between June 2017 and June 2022, including patients diagnosed with asymptomatic carotid webs via CT angiography (CTA), to evaluate the efficacy of antiplatelet therapy as primary prevention of stroke. The primary outcome was the incidence of ischemic stroke. Patients receiving oral anticoagulants or with other etiologies for stroke were excluded. Cox regression with Firth’s penalized maximum likelihood method was used to evaluate the association between antiplatelet therapy and stroke. Propensity scores were applied in sensitive analysis.

**Results:**

A total of 105 patients (median age, 61 years; range, 19–80 years), including 53 (50.5%) male patients, were included. Altogether 31 patients received single antiplatelet therapy. During a median follow-up of 31 months (range from 9 to 89 months), ischemic stroke occurred in 3 of 31 patients (incidence rate, 3.51 per 100 person-years, 95% CI = 0.72–10.25) in the antiplatelet group and 10 of 74 patients (5.16 per 100 person-years, 95% CI = 2.18–9.50) in the non-antiplatelet group. The estimated association favored antiplatelet therapy but was imprecise (HR = 0.755, 95% CI = 0.215–2.653, *p* = 0.642). Similar results were observed after adjustment for atherosclerotic cardiovascular disease (ASCVD) risk and concurrent stenosis, as well as in propensity score-adjusted analyses. Subgroup analysis of patients without high ASCVD risk demonstrated no significant association between antiplatelet therapy and stroke (HR = 0.671, 95% CI = 0.108–4.191, *p* = 0.638).

**Conclusion:**

In this prospective cohort, stroke incidence was numerically lower with single antiplatelet therapy, but estimates were imprecise and compatible with both benefit and harm.

## Introduction

A carotid web is a membrane-like defect that usually originates from the posterior wall of carotid bulb (1). By altering local hemodynamics and inducing blood stasis, carotid webs promote in-situ thrombosis and subsequent distal embolization (2). Although relatively rare, it is an increasingly recognized etiology for cryptogenic stroke, especially in younger patients (3). The prevalence of carotid web was reported to range from 8 to 37% in young patients with cryptogenic stroke (4, 5), compared to less than 1% in non-stroke populations (2, 6). Symptomatic carotid webs have a high stroke recurrence rate of 17 to 56% (7, 8), making secondary stroke prevention essential.

The optimal therapy for carotid web is still unclear (9). Current prevention guidelines recommend antiplatelet therapy for patients with carotid web in the distribution of ischemic stroke or transient ischemic attack (TIA), if no other causes are found, based on case series (10). However, the effect of antiplatelet therapy on asymptomatic carotid webs is unknown. Therefore, we conducted this prospective cohort study to evaluate the efficacy and safety of single antiplatelet therapy as primary prevention of stroke in asymptomatic carotid web.

## Methods

### Participants

We conducted a prospective observational cohort study at the Affiliated Hospital of Qingdao University. Patients were prospectively enrolled between June 2017 and June 2022 after a carotid web was identified on CT angiography (CTA). The clinical indications for CTA included vascular abnormalities detected by ultrasound during annual physical examinations, preoperative assessment, and patients with dizziness, headache, syncope or vertigo. Images of CTA were independently reviewed by two neuroradiologists. A carotid web was defined as a thin, membrane-like filling defect along the posterior wall of the carotid bulb or the origin of the internal carotid artery on oblique sagittal images, with a corresponding thin septum on axial images (5, 11). Patients with no history of cerebral ischemic events and no cerebral infarction on baseline magnetic resonance imaging (MRI) were classified as having asymptomatic carotid webs.

The study was approved by the institutional Ethics committee of the Affiliated Hospital of Qingdao University (no. 2017-102) and conducted in accordance with the Declaration of Helsinki and relevant Chinese regulations on clinical research. Written informed consent to participate was obtained from all participants.

### Study design

The prescription of aspirin was evaluated by the treating physicians based on an individualized clinical assessment, including ASCVD risk, the degree of carotid stenosis, and the clinical assessment of the carotid web. Patients receiving single antiplatelet therapy (aspirin 100 mg/day) were included in antiplatelet cohort, otherwise, they were included in the non-antiplatelet cohort. Patients receiving anticoagulant therapy, switching to carotid revascularization, discontinuing antiplatelet therapy or those with other etiologies of stroke, such as atrial fibrillation, large artery atherosclerosis, cerebral small vessel disease or connective tissue disorders were also excluded. Two patients who underwent carotid revascularization at the time the carotid web was identified were excluded.

Baseline clinical characteristics including age, sex, and atherosclerotic risk factors were extracted from the medical records at enrollment. Dyslipidaemia was defined as elevated total cholesterol, low-density lipoprotein (LDL) cholesterol, or triglycerides, or a low level of high-density lipoprotein (HDL) cholesterol (12). Carotid stenosis were graded using CTA images (13). The 10-year risk for atherosclerotic cardiovascular disease (ASCVD) was assessed according to 2021 European Society of Cardiology (ESC) Guidelines on cardiovascular disease prevention (14).

### Outcomes

Patients were prospectively followed at 3-month intervals by telephone or face-to-face interview. The primary outcome was cerebral ischemic stroke, defined as new-onset neurological deficits with corresponding acute cerebral infarction on MRI in the vascular territory of the ipsilateral carotid web. Follow-up was calculated from the index CTA to the occurrence of the primary outcome, the last documented clinical contact, or the end of the study period, as applicable. Patients who did not experience the primary outcome were censored at their last documented contact. Adverse reaction associated with antiplatelet therapy were also recorded to evaluate safety.

### Statistical analyses

Statistical analyses were conducted using R software (version 4.3.1, Vienna, Austria). Categorical variables were analyzed with the Chi-square test or Fisher’s exact test. Age was summarized as the median with range, and compared by Mann–Whitney U test depending on data distribution. Standardized mean differences (SMD) were calculated to assess baseline covariate imbalances between the two cohorts.

Given the limited sample size and low event rate in this rare disease cohort, we conducted Cox regression with Firth’s penalized maximum likelihood method to evaluate the effect of antiplatelet therapy on stroke (15). Multivariable Cox regression with Firth’s penalized maximum likelihood method was adjusted for ASCVD risk and carotid stenosis. Results were expressed as hazard ratios (HR) with 95% confidence intervals (CIs). To address potential confounding from observed baseline imbalances, we conducted a sensitivity analysis by incorporating a propensity score as an additional covariate in the Firth penalized Cox regression. Propensity scores were estimated using a logistic regression model, incorporating baseline variables selected based on clinical relevance and the magnitude of baseline imbalance. Incidence rates were calculated as events per 100 person-years, with exact 95% confidence intervals computed using the Poisson distribution. To characterize the statistical precision of the survival analysis, the approximate detectable HR was estimated using the Schoenfeld approximation based on the observed number of outcome events and the observed exposure distribution, assuming 80% power and a two-sided α of 0.05. *p* < 0.05 was considered significant.

## Results

A total of 123 patients with asymptomatic web were initially identified. We excluded 14 patients (2 receiving oral anticoagulation, 2 undergoing carotid revascularization, and 10 with other causes of stroke). Of the remaining 109 eligible patients, 4 (3.7%) were lost to follow-up. These four patients were not included in the analysis because reliable follow-up information was insufficient to define a valid censoring time. Ultimately, 105 patients were included in the final analysis. During a median follow-up of 31 months (range from 9 to 89 months), 13 patients (12.4%, 13/105) experienced an ischemic stroke (Figure 1). Clinical and Imaging characteristics of patients with stroke during follow-up were present in Supplementary Table S1. No deaths occurred during follow-up.

**Figure 1:**
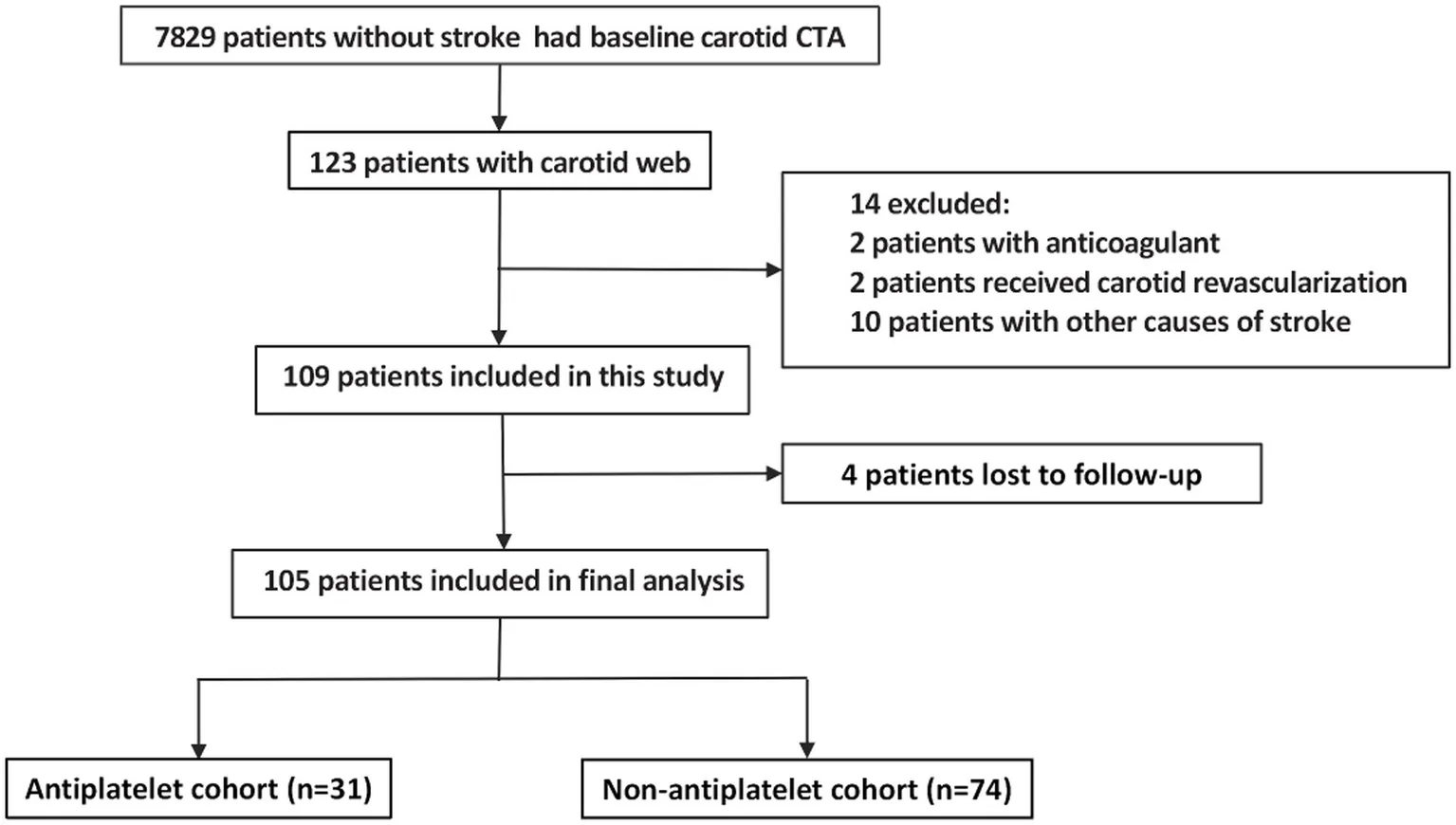
Flow diagram of patient selection.

Baseline clinical characteristics of patients are presented in Table 1. The median age of patients with asymptomatic carotid web was 61 years old (range from 19 to 80 years), with 41.0% (43/105) aged older than 65 years. As for ASCVD risk, 27 patients (25.7%) were classified as high risk, with 8 patients (7.6%) as very high risk. Besides, hypertension was the most common atherosclerotic risk factor (43.8%, 46/105). Only 5 patients (4.8%) were detected with over 50% stenosis of carotid, including 2 patients with over 70% stenosis. Bilateral carotid web were observed in 17 patients (16.2%).

**Table 1:** Comparison of characteristics between antiplatelet and non-antiplatelet cohorts in patients with carotid webs.

Variables	Total (*n* = 105)	Antiplatelet cohort (*n* = 31)	Non-antiplatelet cohort (*n* = 74)	*p* value
Age, median (range), y	61 (19–80)	67 (35–80)	58 (19–80)	0.001
Age ≥65 years	43 (41.0)	20 (64.5)	23 (31.1)	0.001
Male	53 (50.5)	15 (48.4)	38 (51.4)	0.782
Atherosclerotic risk factors				
Diabetes	16 (15.2)	6 (19.4)	10 (13.5)	0.644
Hypertension	46 (43.8)	20 (64.5)	26 (35.1)	0.006
Smoking	19 (18.1)	6 (19.4)	13 (17.6)	0.828
Dyslipidaema	17 (16.2)	8 (25.8)	9 (12.2)	0.083
Statin therapy	15 (14.3)	7 (22.6)	8 (10.8)	0.134
high or very high ASCVD risk	35 (33.3)	18 (58.1)	17 (23.0)	0.001
Carotid artery stenosis ≥ 50%	5 (4.8)	4 (12.9)	1 (1.4)	0.026
Stroke during follow-up	13 (12.4)	3 (9.7)	10 (13.5)	0.826

Variables were present as *n* (%) except age.

### Antiplatelet therapy

Patients in the antiplatelet cohort were significantly older than those in the non-antiplatelet cohort (median age, 67 vs. 58 years, *p* = 0.001, Table 1). Hypertension was more common in the antiplatelet cohort (64.5% vs. 35.1%, *p* = 0.006). In addition, the rate of patients with high or very high ASCVD risk was significantly higher in antiplatelet cohort (58.1% vs. 23.0%, *p* = 0.001).

### Primary prevention of ischemic stroke

During the follow-up, stroke occurred in 3 of 31 patients in the antiplatelet group and 10 of 74 patients in the non-antiplatelet group. The corresponding incidence rates were 3.51 (95% CI = 0.72–10.25) and 5.16 (95% CI = 2.47–9.50) per 100 person-years, respectively. Univariable Firth-penalized Cox regression yielded an HR of 0.755 (95% CI = 0.215–2.653, *p* = 0.642, Figure 2). After adjustment for ASCVD risk and carotid stenosis, the HR was 0.665 (95% CI = 0.167–2.655; *p* = 0.553, Table 2). In the propensity score-adjusted sensitivity analysis, the HR was 0.982 (5% CI = 0.246–3.916, *p* = 0.979, Table 2).

**Figure 2:**
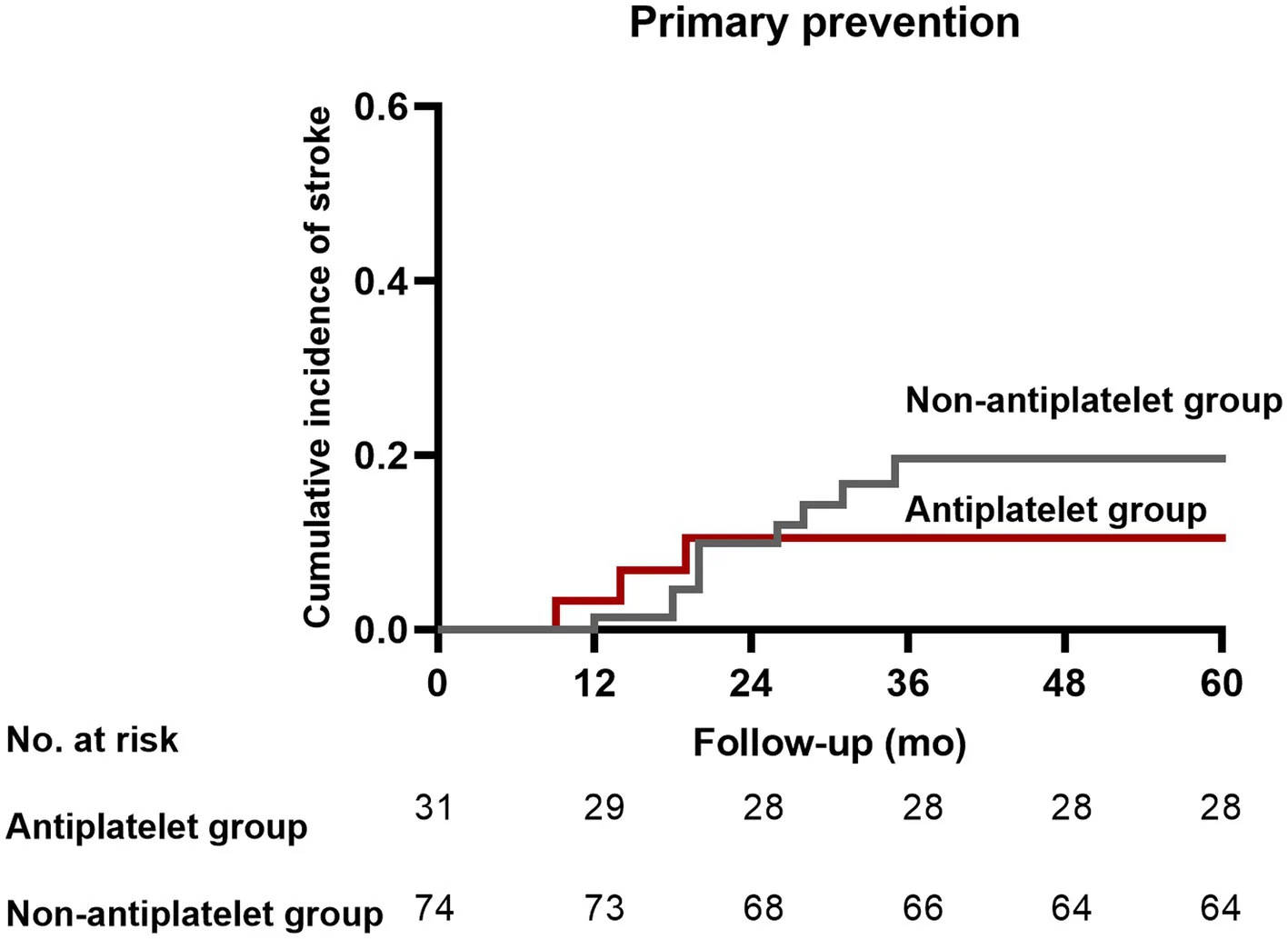
Cumulative incidence curves of stroke in primary prevention. Three patients (3/31, 9.7%) in antiplatelet cohort had an ischemic stroke, compared to 10 of 74 patients (13.5%) in non-antiplatelet cohort (HR = 0.755, 95% CI = 0.215–2.653, *p* = 0.642; adjusted by ASCVD risk and carotid stenosis, HR = 0.665, 95% CI = 0.167–2.655, *p* = 0.553).

**Table 2:** Association between antiplatelet therapy and stroke in asymptomatic carotid webs.

Model	HR	95%CI	*p* value
Univariable Firth Cox	0.755	0.215–2.653	0.642
Adjusted for ASCVD risk and stenosis	0.665	0.167–2.655	0.553
Propensity score-adjusted^a^	0.982	0.246–3.916	0.979
Without high ASCVD risk subgroup	0.671	0.108–4.191	0.638

HR, hazard ratio; CI, confidence interval; ASCVD, atherosclerotic cardiovascular disease. All analyses were conducted by using Firth’s penalized likelihood Cox regression.

a Propensity score derived from age, hypertension, and stenosis.

Regarding adverse effects, one patient reported gingival bleeding, and one patient reported hematochezia. Both patients continued antiplatelet therapy despite these mild bleeding events, and their symptoms resolved within days.

### Primary prevention in patients without high ASCVD risk

In the stratified analysis of 70 patients without high ASCVD risk, 13 patients (18.6%) received antiplatelet therapy. During a median follow-up of 31 months (range from 12 to 66 months), stroke occurred in 1 patient (7.7%, 1/13) in the antiplatelet cohort and 8 patients (14.0%, 8/57) in the non-antiplatelet cohort (Table 3). Consistent with the primary analysis, no significant association was detected between antiplatelet therapy and stroke in this subgroup (HR = 0.671, 95% CI = 0.108–4.191, *p* = 0.638, Table 2).

**Table 3:** Stratified analysis in patients without high ASCVD risk.

Variables	Total (*n* = 70)	Antiplatelet cohort (*n* = 13)	Non-antiplatelet cohort (*n* = 57)	*p* value
Age, median (range), y	58 (19–80)	67 (35–71)	56 (19–80)	0.086
Male	35 (50.0)	6 (46.2)	29 (50.9)	0.759
Atherosclerotic risk factors				
Diabetes	0 (0)	0 (0.0)	0 (0.0)	NA
Hypertension	19 (27.1)	6 (46.2)	13 (22.8)	0.173
Smoking	9 (12.9)	2 (15.4)	7 (12.3)	0.670
Dyslipidaema	9 (12.9)	4 (30.8)	5 (8.8)	0.100
Carotid artery stenosis ≥ 50%	3 (4.3)	2 (15.4)	1 (1.8)	0.086
Stroke during follow-up	9 (12.9)	1 (7.7)	8 (14.0)	0.875

Variables were present as *n* (%) except age. NA, not applicable.

## Discussion

The optimal management of asymptomatic carotid webs remains uncertain. In this prospective cohort study, ischemic stroke occurred less frequently with single antiplatelet therapy, although the association was not statistically significant. Given the small number of outcome events and the wide confidence intervals, the estimate remained compatible with both potential benefit and harm.

The incidence of ipsilateral ischemic stroke in the non-antiplatelet group was 5.16 per 100 person-years. The ipsilateral distribution and isolated or multifocal cortical pattern of all 13 events, together with the exclusion of major competing stroke etiologies, support a thromboembolic mechanism related to the carotid web. This finding raises concern that some patients with asymptomatic carotid web may remain at clinically relevant risk. While current guidelines lack a consensus on the management of asymptomatic carotid webs, surveys indicate that approximately 39% of neurologists and neurointerventionalists prefer antiplatelet therapy for these patients (16).

In this prospective cohort, stroke incidence was numerically lower with single antiplatelet therapy, but estimates were imprecise and compatible with both benefit and harm. Beyond antiplatelet therapy, oral anticoagulation has been considered a theoretical alternative, given the presumed stasis-induced thrombogenesis that similar to atrial fibrillation. Nevertheless, clinical evidence supporting anticoagulant use for asymptomatic carotid webs is currently lacking (9). To eliminate confounding, two patients receiving oral anticoagulants at baseline were explicitly excluded from our cohort. Multi-arm clinical studies are warranted to compare the efficacy and safety of anticoagulants versus antiplatelet agents. Additionally, carotid revascularization has been investigated as a structural treatment for carotid webs, primarily in symptomatic patients. A recent meta-analysis of observational studies indicate that revascularization is associated with a lower risk of recurrent stroke in symptomatic carotid webs, compared to medical management (17). However, these findings cannot be directly extrapolated to asymptomatic carotid webs. Furthermore, while no severe adverse effects were observed in our cohort, the potential hemorrhagic complications of antiplatelet agents should be carefully weighed against their benefits, particularly during long-term primary prevention. Information on longitudinal antihypertensive treatment and blood pressure control was not systematically available, and residual confounding related to concomitant cardiovascular therapy cannot be excluded.

Consistent with previous reports, concurrent carotid stenosis of ≥50% was uncommon in our cohort, occurring in only 4.8% of patients (2). Carotid webs may enlarge the recirculation zone, resulting in disturbed flow and focal blood stasis in the post-web space, which progressively promotes in-situ thrombosis (18). Based on this hemodynamic mechanism, risk stratification using specific anatomic and angioarchitectural features may facilitate individualized treatment. Previous cross-sectional studies indicate that specific morphological parameters, such as the length of the web and its relationship to the carotid bifurcation, are associated with an increased risk of stroke (19–21). However, reliable morphological criteria for risk stratification in asymptomatic carotid web have not been established. Notably, asymptomatic re-thrombosis in patients with carotid web-related stroke indicated that structured imaging surveillance may be useful in stroke prevention (22).

Our study has several limitations. First, based on the 13 observed events and the observed distribution of antiplatelet exposure, the approximate detectable HR at 80% power and a two-sided α of 0.05 was ≤0.18 or ≥5.49, indicating that only very large treatment effects could have been reliably detected. Therefore, the absence of statistical significance should not be interpreted as evidence of no treatment effect. Larger multicenter studies with more outcome events are needed to provide more precise estimates. Second, the non-randomized observational design introduces inherent confounding by indication, as patients with higher ASCVD risk or concurrent mild stenosis were more likely to be prescribed single antiplatelet therapy. While multivariable Cox regression with Firth’s penalized maximum likelihood method, propensity score-adjusted sensitivity analyses, and subgroup analyses yielded consistent results, unmeasured confounding cannot be entirely excluded. Third, we did not stratify the effect of antiplatelet therapy based on detailed anatomic and angioarchitectural features, which requires further investigation. Forth, subclinical thrombus was not evaluated by duplex or CTA during follow-up, which may underestimate the event burden. Finally, considering the presumed lifelong cumulative stroke risk associated with carotid webs, our median follow-up of 31 months may be insufficient to fully capture long-term clinical outcomes.

## Conclusion

In this prospective cohort study, stroke incidence was numerically lower with single antiplatelet therapy, but estimates were imprecise and compatible with both benefit and harm. Further large-scale, multicenter, randomised controlled trials are warranted to establish optimal management guidelines for asymptomatic webs.

## Data Availability

The original contributions presented in the study are included in the article/Supplementary material, further inquiries can be directed to the corresponding author.

## References

[1] Momose KJ New PF. Non-atheromatous stenosis and occlusion of the internal carotid artery and its main branches. Am J Roentgenol Radium Therapy, Nucl Med. (1973) 118:550–66. doi: 10.2214/ajr.118.3.550, 4198748

[2] Chen H Colasurdo M Costa M Nossek E Kan P. Carotid webs: a review of pathophysiology, diagnostic findings, and treatment options. J Neurointerv Surg. (2024) 16:1294–9. doi: 10.1136/jnis-2023-021243, 38290814

[3] Mac Grory B Emmer BJ Roosendaal SD Zagzag D Yaghi S Nossek E. Carotid web: an occult mechanism of embolic stroke. J Neurol Neurosurg Psychiatry. (2020) 91:1283–9. doi: 10.1136/jnnp-2020-323938, 33004431

[4] Joux J Boulanger M Jeannin S Chausson N Hennequin JL Molinie V . Association between carotid bulb diaphragm and ischemic stroke in young afro-Caribbean patients: a population-based case-control study. Stroke. (2016) 47:2641–4. doi: 10.1161/STROKEAHA.116.013918, 27625379

[5] Coutinho JM Derkatch S Potvin AR Tomlinson G Casaubon LK Silver FL . Carotid artery web and ischemic stroke: a case-control study. Neurology. (2017) 88:65–9. doi: 10.1212/WNL.0000000000003464, 27864523 PMC5200857

[6] Kim SJ Allen JW Bouslama M Nahab F Frankel MR Nogueira RG . Carotid webs in cryptogenic ischemic strokes: a matched case-control study. J Stroke Cerebrovasc Dis. (2019) 28:104402. doi: 10.1016/j.jstrokecerebrovasdis.2019.104402 31563567

[7] Zhang AJ Dhruv P Choi P Bakker C Koffel J Anderson D . A systematic literature review of patients with carotid web and acute ischemic stroke. Stroke. (2018) 49:2872–6. doi: 10.1161/strokeaha.118.021907, 30571430

[8] Guglielmi V Compagne KCJ Sarrami AH Sluis WM van den Berg LA van der Sluijs PM . Assessment of recurrent stroke risk in patients with a carotid web. JAMA Neurol. (2021) 78:826–33. doi: 10.1001/jamaneurol.2021.1101, 33970205 PMC8111564

[9] Kim SJ Nogueira RG Haussen DC. Current understanding and gaps in research of carotid webs in ischemic strokes: a review. JAMA Neurol. (2019) 76:355–61. doi: 10.1001/jamaneurol.2018.3366, 30398546

[10] Kleindorfer DO Towfighi A Chaturvedi S Cockroft KM Gutierrez J Lombardi-Hill D . 2021 guideline for the prevention of stroke in patients with stroke and transient ischemic attack: a guideline from the American Heart Association/American Stroke Association. Stroke. (2021) 52:e364–467. doi: 10.1161/STR.0000000000000375, 34024117

[11] Barreau X Holtmannspoetter M Spelle L Herbreteau D Fiehler J Costalat V . Prevalence and site of predilection of carotid webs focusing on symptomatic and asymptomatic Japanese patients. J Neurointerv Surg. (2021) 135:1370–6. doi: 10.3171/2020.8.JNS201727 33668027

[12] Pirillo A Casula M Olmastroni E Norata GD Catapano AL. Global epidemiology of dyslipidaemias. Nat Rev Cardiol. (2021) 18:689–700. doi: 10.1038/s41569-021-00541-4, 33833450

[13] De Rango P Brown MM Leys D Didier L Howard VJ Moore WS . Management of carotid stenosis in women: consensus document. Neurology. (2013) 80:2258–68. doi: 10.1212/WNL.0b013e318296e952, 23751919 PMC3721103

[14] Visseren FLJ Mach F Smulders YM Carballo D Koskinas KC Back M . 2021 ESC guidelines on cardiovascular disease prevention in clinical practice. Eur Heart J. (2021) 42:3227–337. doi: 10.1093/eurheartj/ehab484, 34458905

[15] Lin IF Chang WP Liao YN. Shrinkage methods enhanced the accuracy of parameter estimation using cox models with small number of events. J Clin Epidemiol. (2013) 66:743–51. doi: 10.1016/j.jclinepi.2013.02.002, 23566374

[16] Wojcik K Milburn J Vidal G Tarsia J Steven A. Survey of current management practices for carotid webs. Ochsner J. (2019) 19:296–302. doi: 10.31486/toj.18.0114, 31903051 PMC6928665

[17] Khan F Rehman CA Fletcher L Fayad F Chahien D Akhai A . Carotid revascularization versus medical Management for Ischemic Stroke with ipsilateral carotid web: a systematic review and Meta-analysis. Ann Neurol. (2025) 98:625–33. doi: 10.1002/ana.27280, 40503762

[18] Compagne KCJ Dilba K Postema EJ van Es A Emmer BJ Majoie C . Flow patterns in carotid webs: a patient-based computational fluid dynamics study. AJNR Am J Neuroradiol. (2019) 40:703–8. doi: 10.3174/ajnr.A6012 PMC7048501 30872422

[19] Tabibian BE Parr M Salehani A Mahavadi A Rahm S Kaur M . Morphological characteristics of symptomatic and asymptomatic carotid webs. J Neurosurg. (2022) 137:1727–32. doi: 10.3171/2022.2.JNS212310 35426815

[20] Bala F Alhabli I Singh N Benali F Coutts S Goyal M . Relationship between carotid web morphology on CT angiography and stroke: a pooled multicenter analysis. Int J Stroke. (2024) 19:1046–52. doi: 10.1177/17474930241264141, 38877750

[21] Negash B Wiggan DD Grin EA Sangwon KL Chung C Gutstadt E . Use of carotid web angioarchitecture in stratification of stroke risk. J Neurointerv Surg. (2025) 18:1196–201. doi: 10.1136/jnis-2025-023368 40541402

[22] Ros-Arlanzón P Corona-García DJ Warnken-Miralles MD Ginés-Murcia E Moreno-Navarro L Hernández-Lorid R . Silent threats: asymptomatic re-thrombosis in carotid web and the window for preventive intervention. Neurointervention. (2025) 20:122–9. doi: 10.5469/neuroint.2025.00682, 41033686 PMC12585938

