# Supplementary material for: Antiplatelet therapy for primary prevention of stroke in patients with carotid web: a prospective cohort study
Source: Front Neurol. 2026 Oct 7;17:1955675. doi: 10.3389/fneur.2026.1955675 (PMC13683218; doi:10.3389/fneur.2026.1955675)
Supplement: Supplementary file 1 [file Table_1.docx]

**Table S1 Clinical and Imaging characteristics of patients with stroke during follow-up**

| **Patient ID** | **Age** | **Sex** | **Antiplatelet therapy** | **Time to stroke** | **Laterality of carotid webs** | **Infarct side** | **Stroke subtype** | **ASCVD risk** | **Carotid stenosis ≥50%** | **Imaging characteristics of stroke** |
| --- | --- | --- | --- | --- | --- | --- | --- | --- | --- | --- |
| 1 | 72 | F | Yes | 9m | Right | Right | Cryptogenic, non-lacunar | Very high | Yes | Territorial infarction pattern |
| 2 | 72 | F | No | 28m | Left | Left | Cryptogenic, non-lacunar | Low | No | Cortical infarction involving 1 subdivision |
| 3 | 70 | M | No | 26m | Right | Right | Cryptogenic, non-lacunar | High | No | Territorial infarction pattern |
| 4 | 49 | M | No | 20m | Bilateral | Right | Cryptogenic, non-lacunar | Low | No | Territorial infarction pattern |
| 5 | 51 | M | No | 20m | Left | Left | Cryptogenic, non-lacunar | High | No | Territorial infarction pattern |
| 6 | 33 | M | No | 18m | Right | Right | Cryptogenic, non-lacunar | Low | No | Territorial infarction pattern |
| 7 | 39 | M | No | 31m | Left | Left | Cryptogenic, non-lacunar | Low | No | Multiple small cortical infarcts |
| 8 | 44 | F | No | 35m | Left | Left | Cryptogenic, non-lacunar | Low | No | Multiple small cortical infarcts |
| 9 | 74 | F | Yes | 19m | Right | Right | Cryptogenic, non-lacunar | High | No | Territorial infarction pattern |
| 10 | 58 | M | No | 20m | Left | Left | Cryptogenic, non-lacunar | Low | No | Territorial infarction pattern |
| 11 | 31 | M | No | 18m | Left | Left | Cryptogenic, non-lacunar | Low | No | Territorial infarction pattern |
| 12 | 66 | M | No | 12m | Left | Left | Cryptogenic, non-lacunar | Low | No | Territorial infarction pattern |
| 13 | 47 | M | Yes | 14m | Right | Right | Cryptogenic, non-lacunar | Low | No | Territorial infarction pattern |
